# Homology between SARS CoV-2 and human proteins

**DOI:** 10.1038/s41598-021-96233-7

**Published:** 2021-08-25

**Authors:** Vladimir Khavinson, Alexander Terekhov, Dmitry Kormilets, Alexander Maryanovich

**Affiliations:** 1 Mechinkov North-Western State Medical University, 47 Piskaryovsky Prosp., 195067 St. Petersburg, Russia; 2 Kirov Military Medical Academy, St. Petersburg, Russia; 3 Saint Petersburg Institute of Bioregulation and Gerontology, St. Petersburg, Russia

**Keywords:** Infection, Computational biology and bioinformatics, SARS-CoV-2

## Abstract

An extremely high contagiousness of SARS CoV-2 indicates that the virus developed the ability to deceive the innate immune system. The virus could have included in its outer protein domains some motifs that are structurally similar to those that the potential victim's immune system has learned to ignore. The similarity of the primary structures of the viral and human proteins can provoke an autoimmune process. Using an open-access protein database Uniprot, we have compared the SARS CoV-2 proteome with those of other organisms. In the SARS CoV-2 spike (S) protein molecule, we have localized more than two dozen hepta- and octamers homologous to human proteins. They are scattered along the entire length of the S protein molecule, while some of them fuse into sequences of considerable length. Except for one, all these n-mers project from the virus particle and therefore can be involved in providing mimicry and misleading the immune system. All hepta- and octamers of the envelope (E) protein, homologous to human proteins, are located in the viral transmembrane domain and form a 28-mer protein E_14-41_ VNSVLLFLAFVVFLLVTLAILTALRLCA. The involvement of the protein E in provoking an autoimmune response (after the destruction of the virus particle) seems to be highly likely. Some SARS CoV-2 nonstructural proteins may also be involved in this process, namely ORF3a, ORF7a, ORF7b, ORF8, and ORF9b. It is possible that ORF7b is involved in the dysfunction of olfactory receptors, and the S protein in the dysfunction of taste perception.

## Introduction

The interaction of SARS CoV-2 with the host immune system is largely determined by the structural similarities between viral and host proteins. The studies of SARS CoV-2 are still focused on the S protein^1^.

An extremely high contagiousness of the coronavirus SARS CoV-2 indicates that during its evolution the virus developed the ability to deceive the innate immune system. The simplest way to achieve this ability would be to incorporate into its membrane the proteins that share structural similarity with those which the immune system of the potential victim has learnt to ignore. Probably, the virus borrowed some n-mers from bats or other mammals. Any motif of any mammalian protein was suitable for borrowing, if only the immune system considered it to be of its own.

The knowledge of the homology between the SARS CoV-2 and human proteins would help understand the mechanisms of mimicry at the moment of infection. The SARS CoV-2 proteins may simulate human proteins, mislead the immune system, and slow down its response.

However, mimicry is not the only process that is determined by the protein homology between the virus and host organism. After the inevitable destruction of the virus particle, the proteins or their domains, which were inside the virus until then, come into contact with the immune system. With some structural similarity, a part of the immune response will be directed against the proteins of the host organism, i.e., an autoimmune response will arise.

This study aimed to identify the human proteins which share a significant structural homology with the SARS CoV-2 proteins. We hope this information will be useful to the developers of vaccines against coronavirus.

Joshua Lederberg^2^ believed that "microbes and their human hosts constitute a *superorganism*." According to this, we considered the concept of "human proteins" as a combination of human own proteome and the proteomes of gut microbiota. We have paid particular attention to the proteins that are involved in the three functions that are almost necessarily affected in this disease, namely digestion, olfaction and taste.

## Methods

Using an open-access protein database Uniprot and our original computer program Ouroboros^3^, we compared the SARS CoV-2 proteome^4^ with those of other organisms. We also searched for a separate database of 75,777 human proteins^5^. The algorithm we used compares primary sequences of SARS CoV-2 and human proteins, presented in the form of a one-letter code. We performed a comparison of proteins by a consecutive search for regions of one protein in the others, which is essentially a standard task of finding a substring in a string. This algorithm is implemented in standard methods of many programming languages, including Python, in which the main program was coded. The URL to the source code is provided above^3^.

When assessing the homology between the viral and human proteins, we took into account the presence of the common 7-/8-mers and especially their fusion into longer sequences. For example, 7-dimensional viruses, one of which is homologous to the human protein A, and the other to the protein B, can "overlap" at the ends, forming regions of 8 to 14 amino acid residues in length.

## Results and discussion

### Structural proteins

#### Spike glycoprotein

##### S protein, 1273 aa


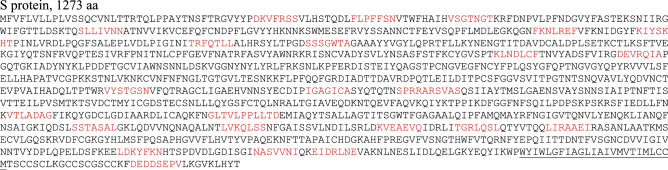


Hereinafter, regions homologous to human proteins are highlighted in red. Transmembrane tail TM_1214-1237_ is underlined.

In the S protein molecule, we localized more than two dozen of 7-/8-mers homologous to human proteins (Table 1).

**Table 1:** Localization of homologous 7-/8-mers in the S protein and human proteins.

Subunit	SARS CoV-2 S protein domain	In S protein	In human proteins
S1	Signal peptide (N-terminus)_1–13_	None	–
	N-terminus domain NTD_14-305_	DKVFRSS_40-46_	Zinc finger protein 528_275–281_
		FLPFFSN_55-61_	OTU domain-containing protein 6A_185-191_
		VSGTNGT_70-76_	Lysosome-associated membrane glycoprotein 1_171–177_
		SLLIVNN_116-122_	ATP-binding cassette sub-family A member 10_825–831_
		FKNLREF_186-192_	Isovaleryl-CoA dehydrogenase, mitochondrial_77-83_
		TRFQTLL_236-242_	Disheveled-associated activator of morphogenesis 2_251–257_
		KIYSKHT_202-208_	Uncharacterized protein C1orf105_7-13_
		SSSGWTA_254-260_	Uncharacterized protein KIAA1109 (Fragment)_610–616_
	Uncharacterized fragment_306-318_	None	—
	Receptor-binding domain RBD_319-541_	KLNDLCF_386-392_	Interleukin-7_149–155_
		DEVRQIA_405-411_	Histone-lysine N-methyltransferase 2C_4530-4536_
	Uncharacterized fragment_542-787_	VYSTGSN_635-641_	Neural cell adhesion molecule L1-like protein_341-347_
		IGAGICA_666-672_	Hepatitis A virus cellular receptor 2_205–211_
		SPRRARS_680-686_	Hermansky-Pudlak syndrome 1 protein_258-264_
		RRARSVAS_682-689_	Amiloride-sensitive sodium channel subunit alpha_201-208_
S2	Fusion peptide FP_788-806_	None	—
	Uncharacterized fragment_807-911_	VTLADAG_826-832_	Non-receptor tyrosine-protein kinase TNK1_440-446_
		GLTVLPP_857-863_	FH1/FH2 domain-containing protein 3_972–978_
		LPPLLTD_861-867_	Maestro heat-like repeat-containing protein family member 9_250–256_
	Heptapeptide repeat sequence 1 HR1_912-984_	SSTASAL_939-945_	40S ribosomal protein S13_143-149_
		LVKQLSS_962-968_	E3 SUMO-protein ligase PIAS1_284-290_
	Uncharacterized fragment_985-1162_	KVEAEVQ_986-974_	Emilin-3_625–631_
		TGRLQSL_998-1004_	Neuron navigator 3_1610–1616_
		LIRAAEI_1012-1018_	Unconventional myosin-XVIIIa_1352-1358_
		LDKYFKN_1152-1158_	Follistatin-related protein 1_149–155_
	Heptapeptide repeat sequence 2 HR2_1163-1213_	NASVVNI_1173-1179_	Thyroid adenoma-associated protein_1022-1028_
		EIDRLNE_1182-1188_	Protein SETSIP_64-70_; Protein SET_54-60_
	Transmembrane tail TM_1214-1237_	None	–
	Cytoplasm tail CT_1238-1273_	DEDDSEPV_1257-1264_	Unconventional myosin-XVI_1404-1421_

Fragments homologous to human proteins are scattered along the entire length of the S protein molecule, and some of them fuse in sequences of considerable length, namely 10-mers SPRRARSVAS_680-689_, 11-mers GLTVLPPLLTD_857-867_ and two closely spaced 7-mers NASVVNI_1173-1179_ and EIDRLNE_1182-1188_. Octamer RRARSVAS_682-689_ is located at the junction of the S1 and S2 subunits. All these n-mers stand out from the virus particles and may be involved in the effect of mimicry.

SARS CoV-2 can cause smell and taste dysfunction, as well as muscle injury^6^.

The 8-mer DEDDSEPV_1257-1264_, located in the cytoplasmic tail, can be released during the destruction of the virus particle and get involved in orchestrating the immune system’s response, directing a part of it to the homologous 8-mer in human unconventional myosin-XVI_1404-1421_. The role of this mechanism in muscle dysfunction in coronavirus infection deserves a special investigation.

The 8-mer RRARSVAS_682-689_ is homologous to the amiloride-sensitive sodium channel subunit alpha_201-208_, which is involved in salt taste perception^7^.

With a high degree of probability, it can be argued that the S protein is involved in the process of mimicry. It may also take some part in provoking an autoimmune response.

We have checked the S protein homology across10 species, specifically primates, bats and some other mammals. The results are presented in Table entitled *Similarity of SARS CoV-2 spike glycoprotein structure with some mammalian proteins* in the electronic attachement. Probably, attention should be paid to the homologous regions common to SARS CoV-2, humans, and bats. The data presented so far do not allow us to derive a more general rule.

#### Envelope small membrane protein

##### E protein, 75 aa (transmembrane domain_8-38_ is underlined)


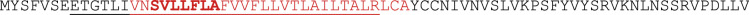


In the E protein molecule, we localized seven 7-mers and one 8-mer homologous to human proteins (Table 2).

**Table 2:** Localization of homologous 7-/8-mers in the E protein and human proteins.

E protein domains^a^	In E protein	In human proteins
Signal peptide (N-terminus domain)_1–7_	None	–
Transmembrane domain_8-38_	VNSVLLF_14-20_	Heterogeneous nuclear ribonucleoprotein L_191-197_
	VNSVLLFL_14-21_	Ran-binding protein 6_409–416_
	NSVLLFL_15-21_	Lysosomal amino acid transporter 1 homolog_133-139_
	SVLLFLA_16-22_	Cytochrome P450 2B6_4-10_ ; Cytochrome P450 2B7_4-10_; GPI ethanolamine phosphate transferase 3_5–11_
	LAFVVFL_21-27_	Solute carrier family 15 member 4_235–241_
	VFLLVTL_25-31_	Alpha-(1,3)-fucosyltransferase 10_20–26_
	LAILTAL_31-37_	Transient receptor potential cation channel subfamily M member 6_394–400_ ; Transient receptor potential cation channel subfamily M member 3_465–471_
	TALRLCA_35-41_^b^	Protein disulfide-isomerase TMX3_8-14_
Internal domain_39-75_	None	–

^a^Domain boundaries see in^8^.

^b^Heptamer TALRLCA_35-41_ is located at the junction of the transmembrane domain_8-38_ and internal domain_39-75_.

A fragment of the E_8-38_ protein transmembrane domain can be represented as follows:
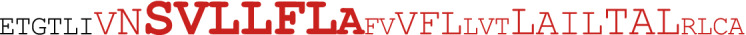


The size of the letters (point size) corresponds to the frequency of the viral 7-/8-mers in the human proteome.

The protein E transmembrane domain contains 7-/8-mers, homologous to the proteins of some gut bacteria and even cereals, for example, corn, sorghum, wheat, and barley (Table 3).

**Table 3:** Localization of some of homologous 7-/8-mers in the E protein and human gut proteome.

In E protein	In bacterial and plant proteins
**A**FVVFLLV_22-29_	Lpp126 large-conductance mechanosensitive channel: Lactobacillus casei_80-87_; L. paracasei_80-87_; L. florum_80-87_
TLAILTA_30-36_	Uncharacterized proteins: Zea mays_90-164_; Sorghum bicolor_97-127_; Triticum aestivum_116-190_; Hordeum vulgare_87-161_

The simulation targets may have been the proteins synthesized by a macroorganism itself or by its normal gut microbiota.

All protein E 7-/8-mers, homologous to proteins of humans, gut bacteria and cereals, are located in the transmembrane domain of the virus and form the 28-mer protein E_14-41_. A random selection of 28 amino acid residues in a row would require an astronomical number of iterations: 20^28^ = 2.7 ∙ 10^36^.

The involvement of the E protein in mimicry is hardly possible, but its implication in provoking an autoimmune response (after the destruction of the virus particle) seems very likely.

As a major target, the viral E protein has usually been used for the development of vaccines, specifically against HIV-1^9^, Dengue virus^10^, hepatitis B virus^11^, SARS CoV-2^12^ and many other viruses. A deletion of the SARS-CoV E protein reduces pathogenicity and mortality in laboratory animals^13^. In the transmembrane domain of the SARS-CoV E protein, specific critical virulence-determining features have been identified^14^.

#### Membrane protein

##### Membrane protein, 222 aa


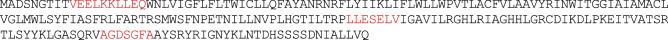


In the M protein molecule, we localized six 7-mers homologous to human proteins (Table 4).

**Table 4:** Localization of homologous 7-mers in the M protein and human proteins.

In M protein	In human proteins
VEELKKL_10-16_	Glutaredoxin-related protein 5, mitochondrial_135-141_
EELKKLL_11-17_	GDP-fucose protein O-fucosyltransferase 2_340–346_
ELKKLLE_12-18_	Cullin-1_335–341_
LKKLLEQ_13-19_	Filamin-A-interacting protein 1_211–217_
LLESELV_133-139_	Leucine-rich repeat-containing protein 71_439–445_
AGDSGFA_188-194_	Myosin-14_359–365_

A N-terminus fragment_1-19_ of the M protein can be represented as follows:
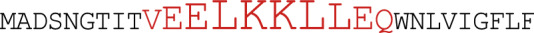


In the protein M, four 7-dimensional homologues of human proteins are fused into 10-mer VEELKKLLEQ_10-19_, the hydrophilic composition of which indicates a possible contact with the external environment, i.e., with the host's immune system, and the involvement in mimicry.

Outside of the 10-mer, we found only two homologous 7-mers. It is unlikely that the M protein is involved in provoking an autoimmune response (after the destruction of the virus particle).

#### Nucleoprotein

##### Nucleoprotein, 419 aa


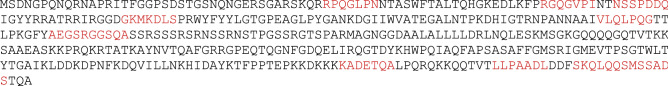


In the N protein molecule, we localized eleven 7-mers homologous to human proteins (Table 5).

**Table 5:** Localization of homologous 7-mers in the N protein and human proteins.

In N protein	In human proteins
RPQGLPN_41-47_	GATOR complex protein WDR59_757-763_
RGQGVPI_68-74_	Putative uncharacterized protein encoded by LINC00346_154-160_
NSSPDDQ_77-83_	NEDD4-binding protein 2_154–160_
GKMKDLS_99-105_	Chromodomain-helicase-DNA-binding protein 1-like_770-776_
VLQLPQG_157-163_	Prestin_92-98_
AEGSRGG_173-179_	snRNA-activating protein complex subunit3_2-8_
SRGGSQA_176-182_	Ras-associating and dilute domain-containing protein_886-892_
KADETQA_375-381_	Myopalladin_90-96_
LLPAADL_394-400_	Probable E3 ubiquitin-protein ligase HERC1_1098-1104_
SKQLQQS_404-410_	Codanin-1_259–265_
SMSSADS_410-416_	Protein PRRC2B_416-422_

The N protein is located completely inside the virus particle and cannot be involved in mimicry. All heptamers homologous to human proteins form several rather long fragments, including the 13-mer SKQLQQSMSSADS_404-416_ and 10-mer AEGSRGGSQA_173-182_, which increases the likelihood of the protein involvement in provoking an autoimmune response.

### Nonstructural proteins

All non-structural proteins of SARS CoV-2 are located completely inside the virus particle and, by definition, cannot be involved in the process of mimicry. It remains to consider the possibility of their implication in provoking an autoimmune process.

#### ORF3a protein

##### ORF3a protein, 275 aa


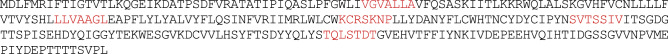


In the ORF3a protein molecule, we localized five 7-mers homologous to human proteins (Table 6).

**Table 6:** Localization of homologous 7-mers in the ORF3a protein and human proteins.

In ORF3a protein	In human proteins
VGVALLA_48-54_	Manganese-transporting ATPase 13A1_876-882_
LLVAAGL_95-101_	Glycerophosphoinositol inositolphosphodiesterase GDPD2_129-135_
KCRSKNP_132-138_	Vacuolar protein sorting-associated protein 13A_2066-2972_
SVTSSIV_162-168_	Protein piccolo_2779-2785_
TQLSTDT_217-223_	Septin-14_418–424_

The 7-mers scattered along the entire length of its molecule do not form long n-mers anywhere else. ORF3a does not appear to be involved in provoking an autoimmune response.

#### ORF7a protein

##### ORF7a 121 aa


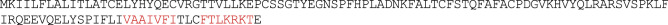


In the ORF7a protein molecule, we found two 7-mers homologous to human proteins and located in close proximity to each other (Table 7).

**Table 7:** Localization of homologous 7-mers in the ORF7a protein and human proteins.

In ORF7a protein	In human proteins
VAAIVFI_104-110_	Transmembrane protein 255B_86-92_
FTLKRKT_114-120_	Cytosolic 5'-nucleotidase 3A_36-42_

It is possible that ORF7a is involved in provoking an autoimmune response.

#### ORF7b protein

##### ORF7b protein, 43 aa


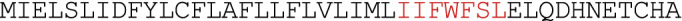


In this polypeptide, we found only one 7-mer homologous to the human protein (Table 8).

**Table 8:** Localization of the homologous 7-mer in ORF7b and a human protein.

In ORF7b protein	In human protein
IIFWFSL_26-32_	Olfactory receptor 7D4_151-157_

ORF7b may be involved in provoking an autoimmune response, contributing to olfactory dysfunction.

#### ORF8 protein

##### ORF8 protein, 121 aa


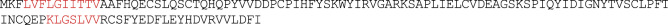


The primary structure of SARS-CoV-2 ORF8 is close to that of bat RaTG13-CoV^15^. In this polypeptide, there are three 7-mers homologous to human proteins (Table 9).

**Table 9:** Localization of homologous 7-mers in the ORF8 protein and human proteins.

In ORF8 protein	In human proteins
LVFLGII_4-10_	Zinc finger protein 486_49–55_
LGIITTV_7-13_	D-2-hydroxyglutarate dehydrogenase, mitochondrial_262-268_
KLGSLVV_94-100_	Sodium leak channel non-selective protein_505-511_

Due to the fusion of two 7-mers into 10-mer LVFLGIITTV_4-13_, the ORF8 protein can be involved in provoking an autoimmune response.

#### ORF9b protein

##### ORF9b protein, 97 aa


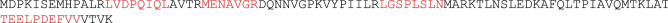


In the ORF9b protein molecule, we localized six 7-/8-mers, homologous to human proteins (Table 10).

**Table 10:** Localization some of homologous 7-/8-mers in ORF9b protein and human proteins.

In ORF9b protein	In human proteins
LVDPQIQL_14-21_	Valine—tRNA ligase, mitochondrial_996-1002_
MENAVGR_26-32_	Neprilysin_419-425_
LGSPLSL_48-54_	Stress-responsive DNAJB4-interacting membrane protein 1_37–43_
GSPLSLN_49-55_	E3 ubiquitin-protein ligase HERC2_4533-4539_
TEELPDE_84-90_	KH homology domain-containing protein 4_465–471_
ELPDEFVV_86-93_	Maestro heat-like repeat-containing protein family member 2B_103-110_

Some of these 7-/8-mers merge into larger n-mers TEELPDEFVV_84-93_ and LGSPLSLN_48-55_.

**Figure 1:**
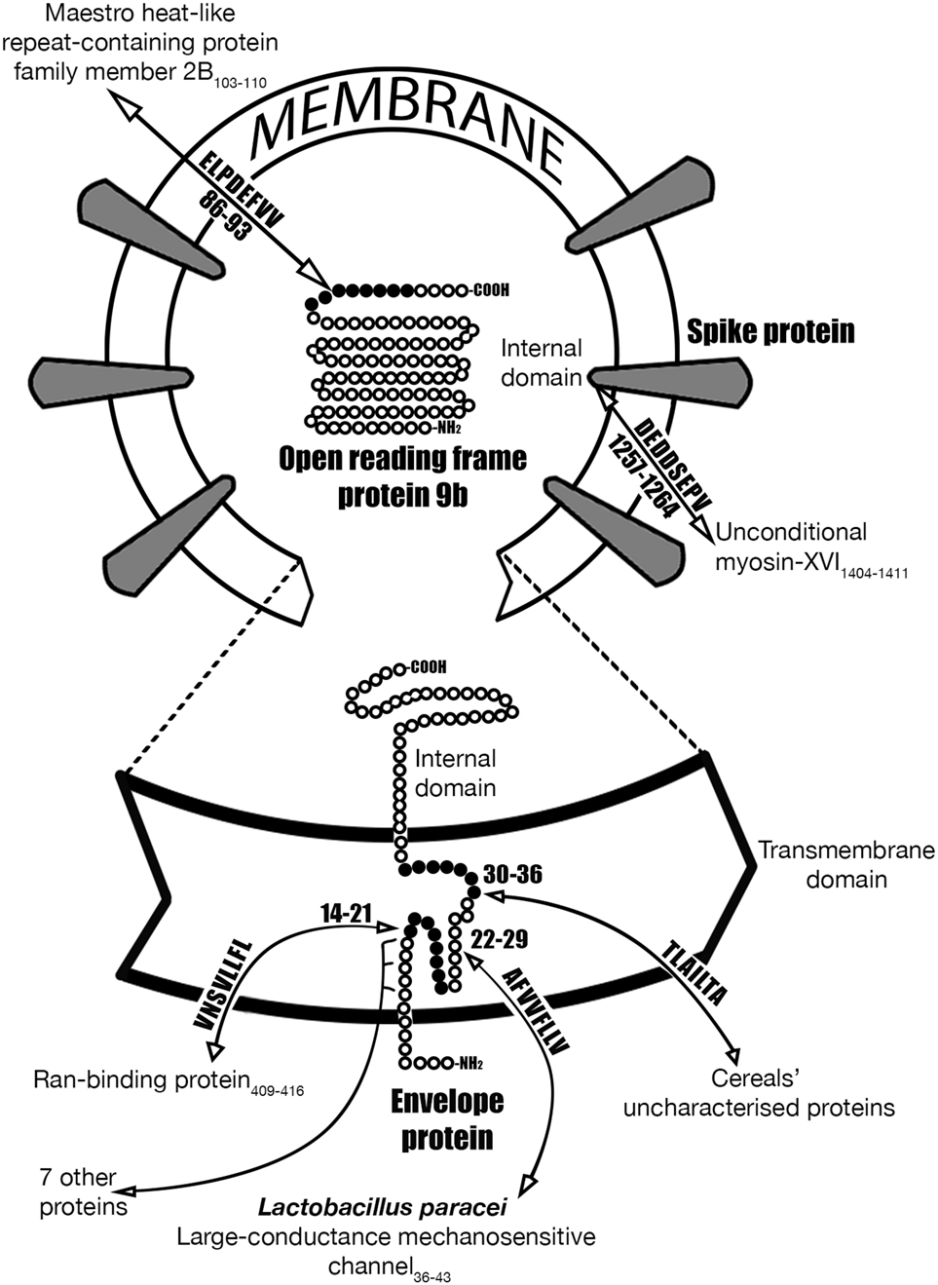
The SARS CoV-2 S, E and ORF9b protein molecules contain hepta/octamers that are homologous to proteins in the human body, including some nutrients and intestinal commensal bacteria.

Octamer ELPDEFVV_86-93_ is homologous to the Maestro heat-like repeat-containing protein family member 2B (Fig. 1), which may play a role in the sperm capacitation^16^. Male reproductive dysfunction was proposed as a likely consequence of COVID-19^17^.

After the destruction of the virus particle, ORF9b can take part in provoking an autoimmune response.

#### Replicase polyprotein RPP 1a

##### Replicase polyprotein RPP 1a, 4405 aa


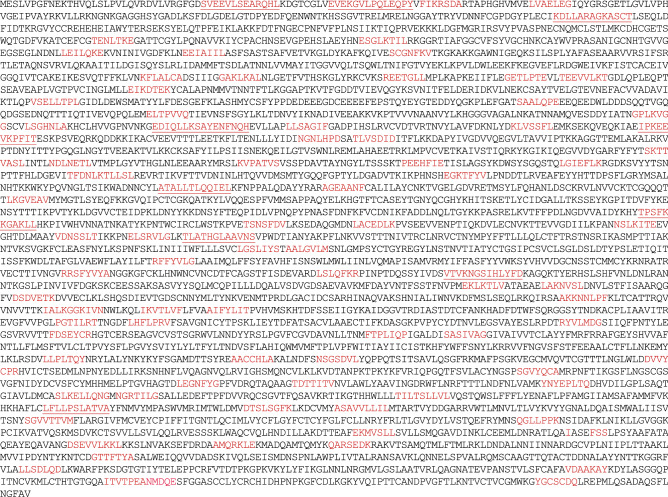


The longest n-mers are underlined.

In the RPP 1a molecule, we localized eleven 8-mers (Table 11) and more than a hundred 7-mers homologous to human proteins.

**Table 11:** Localization of homologous 8-mers in RPP 1a and human proteins.

In Replicase polyprotein 1a	In human proteins
EVEKGVLP_55-62_	Bifunctional heparan sulfate N-deacetylase/N-sulfotransferase 1_214–221_
ESGLKTIL_390-397_	Annexin A7_404-411_
REETGLLM_724-731_	Estrogen-related receptor gamma_30-37_
GGSCVLSG_1100-1107_	Sorting nexin-27_112–119_
DIQLLKSA_1127-1134_	Echinoderm microtubule-associated protein-like 1_38–45_
RRSFYVYA_2431-2438_	Transmembrane protein adipocyte-associated 1_225–232_
AKKNNLPF_2733-2740_	Acyl-CoA:lysophosphatidylglycerol acyltransferase 1_199–206_
YNYEPLTQ_3500-3507_	DNA helicase_199-206_
SLKELLQN_3530-3537_	Centromere protein I_496-503_
DTSLSGFK_3671-3678_	Solute carrier family 12 member 7_995–1002_
PEANMDQE_4312-4319_	Arachidonate 5-lipoxygenase-activating protein_54-61_

Some of the 8-mers are found in more than one human protein, some fold into long n-mers, for example EDIQLLKSAYENFNQH_1126-1141_, EVEKGVLPQLEQPY_55-68_ and SVEEVLSEARQHL_34-46_.

**Figure 2:**
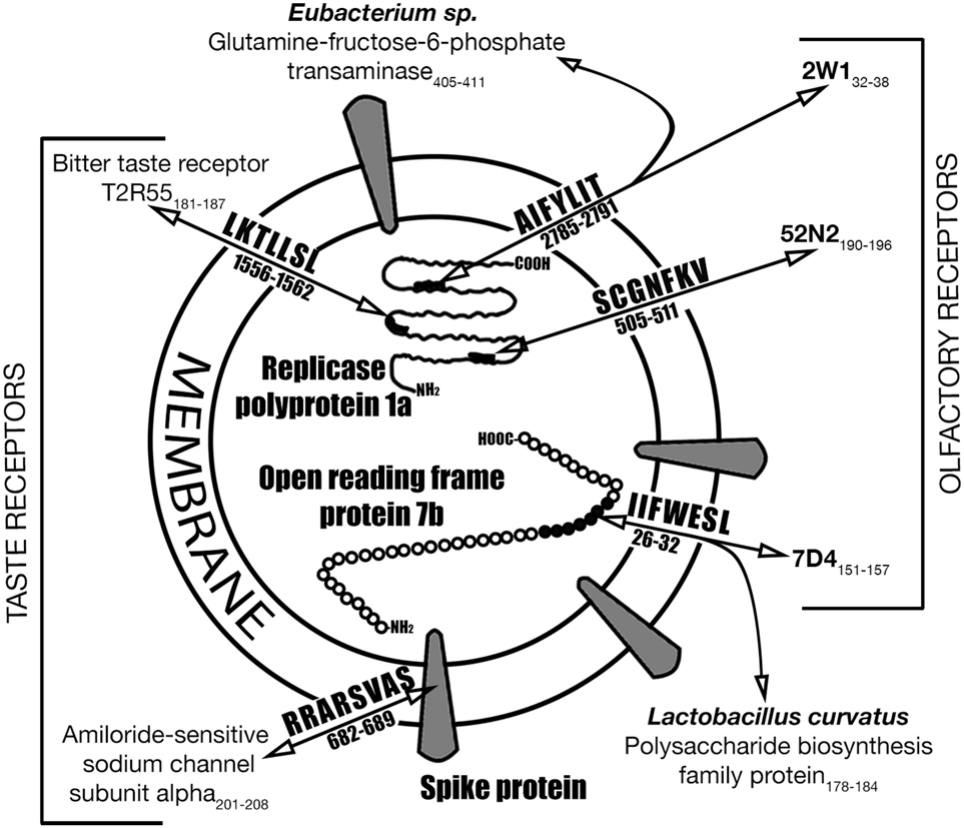
Some SARS CoV-2 hepta/octamers are homologous to human olfactory and taste receptor proteins. Homology to some proteins of commensal gut bacteria is also shown.

In the RPP 1a molecule, 7-mers SCGNFKV_505-511_ and AIFYLIT_2785-2791_ are homologous to human olfactory receptor proteins 52N2_190-196_ and 2W1_32-38_, respectively. A heptamer LKTLLSL_1556-1562_ is homologous to the human bitter taste receptor T2R55_181-187_ (Fig. 2).

#### Replicase polyprotein RPP 1ab

This huge (7096 aa; the primary structure see in^18^) molecule contains 210 hepta- and octamers homologous to human proteins. Some of them fold into long (more than 15 aa) n-mers.

The possibility of the involvement of replicases in provoking an autoimmune response is debatable. Enzymes in general, and cell cycle enzymes in particular, are evolutionarily highly conserved. Fragments homologous to human proteins must be thrown in huge quantities into the gut lumen during the decay of any microorganism that dies there. It is possible that the interaction of replicases with the host's immune system obeys the laws other than for shorter proteins.

#### ORF6, ORF10, and ORF14

In these polypeptides (61, 38, and 73 aa, respectively), we did not find 7-/8-mers homologous to human proteins. When assessing the role of SARS CoV-2 proteins in mimicry and provoking an autoimmune response in humans, we considered the following parameters: (i) the number of homologous n-mers; (ii) the compactness of their arrangement in the SARS CoV-2 protein molecules; (iii) intradomain localization (external, transmembrane, internal) of the SARS CoV-2 proteins, and (iv) physiological functions that involve the homologous human proteins (Table 12).

**Table 12:** Qualitative assessment of the possibility for the SARS CoV-2 proteins to be involved in the processes of mimicry and provoking an autoimmune response.

Goup of proteins	Protein	Mimicry	Autoimmune response	Comment
Structural	S	+++	+	Taste?—Amiloride-sensitive sodium channel subunit alpha_201–208_ Muscle contraction?—Unconventional myosin-XVI_1404–1421_
	E	−	+++	Gut microbiota?—*Lactobacillus paracasei* Digestion?—Cereals’ proteins
	M	++	−	
	N	−	++	
Nonstructural	ORF3a	−	+	
	ORF6	−	−	No homology
	ORF7a	−	+	
	ORF7b	−	+	Smell?—Olfactory receptor 7D4 Gut microbiota?—*Lactobacillus curvatus*
	ORF8	−	++	
	ORF9b	−	++	Sperm capacitation?—Maestro heat-like repeat-containing protein family member 2B_103–110_
	ORF10	−	−	No homology
	ORF14	−	−	No homology
	RPP1a	−	?	Taste?—T2R55 receptor Smell?—Olfactory receptors 2W1 and 52N2 Gut microbiota?—*Eubacterium *sp.
	RPP1ab	−	?	

## Conclusions

Analysis of homology between the SARS CoV-2 and human proteins led us to the following conclusions. Some of the SARS CoV-2 proteins can be implicated in mimicry that can delay the response of innate immunity to the invasion of virus particles into a macroorganism, and in provoking an autoimmune process that directs a part of the immune response to the proteins of a macroorganism (after the destruction of virus particles). Mimicry is probably more characteristic of the spike (S) protein, and the provocation of an autoimmune response seems to be a distinctive feature of the envelope (E) protein. The ORF7b protein may be involved in the impairment of olfactory receptors, and the S protein may be involved in taste perception dysfunction.

Drugs aimed at destructing or blocking these and alike regions in proteins of SARS CoV-2 and other viruses can enable the human immune system not to succumb to viral deception and destroy the invader shortly after its penetration into a macroorganism. It should also be borne in mind that drugs affecting such imitation regions can damage native proteins present of the human body. Destroying or blocking such regions can weaken the autoimmune response.

## Data Availability

The highest.

## Code availability

Source code of Ouroboros (v. 0.5) is fully available at github. URL: https://github.com/liquidbrainisstrain/ouroboros. Artwork: We used GIMP (Version 2.10.22) to create our artwork. The figures are completely original and have not been published anywhere.
